# Mitoxantrone-liposome Sensitizes *FLT3-ITD* Acute Myeloid Leukemia to Gilteritinib Treatment

**DOI:** 10.7150/jca.105557

**Published:** 2025-02-28

**Authors:** Shiyi Yuan, Ying Zhou, Yifei Li, Zhe Chen, Wenrui Xiao, Danqing Jiang, Ping Zhang, Ying Zhang, Fengxia Bai, Jianchuan Deng, Shifeng Lou

**Affiliations:** 1 Department of Hematology, The Second Affiliated Hospital of Chongqing Medical University, Chongqing, Medical University, Chongqing 400000, China.; 2 People's Hospital of Qianxinan Prefecture, Guizhou Province 562400, China

**Keywords:** acute myloid leukemia (AML), *FLT3-ITD*, gilteritinib, mitoxantrone-liposome, combination therapy, cell cycle

## Abstract

FMS-like tyrosine kinase 3 (*FLT3*) is the most frequently mutated gene in acute myeloid leukemia (AML), and is associated with poor prognosis and a high relapse rate. Gilteritinib, a second-generation FLT3 inhibitor, is an important target drug for treating patients with *FLT3*-internal tandem duplication (ITD) AML, is approved for the treatment of relapsed/refractory *FLT3*-mutant acute myeloid leukemia, although challenges such as drug resistance and reduced potency remain. Herein, mitoxantrone-liposomes sensitized *FLT3-ITD* AML cells to gitretinib both *in vivo* and *in vitro*. RNA-sequencing revealed that combination treatment resulted in specific changes in gene expression as well as predicted the mechanism. Primary AML cells harvested from patients with *FLT3-ITD* AML showed a significant response to combination treatment *in vitro.* Our data suggests a novel and promising therapeutic strategy for patients with *FLT3-ITD* AML and relapsed/refractory FTL3-ITD AML.

## Introduction

Acute myeloid leukemia (AML) is a highly heterogeneous hematologic malignancy characterized by clonal proliferation of myeloid cells, and is a common type of adult leukemia 1,2. In 2022, the American Cancer Society estimated that 59,620 new cases of leukemia would be diagnosed in the United States in 2023, with an estimated 23,710 leukemia-related deaths. Historically, this disease has had poor outcomes, with only 40% of young (≤60 years) patients surviving for >5 years 2-4. Mutations in the FMS-like tyrosine kinase 3 (*FLT3*) gene account for approximately 30% of AML cases, and internal tandem duplication (ITD) is the most common type of *FLT3* mutation observed in approximately 25% of those with AML 5. *FLT3* is a transmembrane ligand-activated receptor tyrosine kinase normally expressed in hematopoietic stem and progenitor cells 5. *FLT3* plays an important role in the early stages of myeloid and lymphoid development by promoting cell survival, proliferation, and differentiation through the Phosphatidylinositol-3-kinase (PI3K), RAS, Transcription factor 5 (STAT5), and protein kinase B (AKT) signaling pathways 6. Patients with *FLT3-ITD* mutated AML have a high burden of leukemia and poor prognosis 7. Gilteritinib, a second-generation TKI inhibitor of *FLT3*, cleared the phase 3 clinical trials, was approved for AML therapy, and is widely used in clinical practice 8. It is clinically active against both the inactive and active conformational states of the *FLT3* kinase domain 9. However, a drawback of gilteritinib as a single-agent drug for treatment is the development of acquired drug resistance, involving the reactivation of the PI3K/AKT and/or RAS/MEK/mitogen-activated protein kinase pathways and the continuous expression of *FLT3*-mediated cell transformation-related genes, which increase susceptibility to drug resistance 10, 34. Therefore, while treating *FLT3-ITD* AML, a combination chemotherapy regimen should be administered to reduce resistance.

Mitoxantrone belongs to the anthracycline class of antitumor drugs and is a potent inhibitor of type IIA topoisomerase in tumor cells 11. Owing to its topoisomerase II-mediated mechanism of DNA damage in tumor cells, mitoxantrone has broad antitumor effects 12. Previous studies have identified multiple regulatory mechanisms between mitoxantrone activity and the phosphokinase triodide/protein kinase B/AKT pathway 13,14. Mitoxantrone has been shown to be effective in treating lymphoma and AML alone and in combination with antimetabolic drugs. However, it has some obvious adverse effects resulting in decreased use, especially in older adults with leukemia 15,16.

In this study, we first measured the therapeutic efficacy of mitoxantrone-liposomes in leukemia cell lines and primary AML cells and compared them with those of mitoxantrone *in vitro* and *in vivo*. Mitoxantrone-liposomes were more effective than mitoxantrone, especially in *FLT3-ITD* mutated AML cells and patients. Thus, our data demonstrated that a combination of gilteritinib and mitoxantrone-liposomes enhanced apoptosis and shortened DNA replication (S phase) compared to a single-drug treatment *in vitro* and *in vivo.* In addition, we performed RNA-sequencing (RNA-seq) in MV4-11 cells after 24 h of combination therapy and found that it significantly altered gene expression and decreased drug resistance compared to single drugs. Mechanistically, combination therapy upregulated the expression of genes downstream of p53 signaling and downregulated phosphorylated cyclin dependent kinase inhibitor 1A (CDKN1A) (p21). Combination therapy in the *FlT3-ITD* AML xenograft model resulted in prolonged survival and reduced leukemia burden compared with single drugs. Our study provides a novel high-performance-to-price-ratio combination therapy for clinical treatment of *FLT3-ITD* AML.

## Materials and methods

### Drugs

Gilteritinib (ASP2215), Mitoxantrone (T6588) and Phosphorylation P21 inhibitor (UC2288) (C001) were purchased from Topscience (TargetMpi, Shanghai, China). Stock solutions in dimethyl sulfoxide (DMSO) (Solarbio science and Technology, Beijing, China) were stored at -20°C.

### Cell cultures

The human leukemia cell lines, including THP-1, HL-60, K562, MOLM13, and MV4-11 were purchased from Shanghai Biyuntian Biotechnology Co. Ltd. (Shanghai, China). All cell lines were cultured in Rosewell Park Memorial Institute (RPMI) 1640 medium supplemented with 10% fetal bovine serum, 2 mmol/L L-glutamine, 100 U/mL penicillin and 100 mg/mL streptomycin, and incubated in a humidified 5% CO2/95% air environment at 37 °C. All the cell lines were routinely tested for mycoplasma.

### Clinical samples

*FLT3-ITD* AML samples were obtained from patients treated at The Second Affiliated Hospital of Chongqing Medical University. Normal peripheral blood mononuclear cells (PBMCs) from healthy donors were collected from the hospital at Chongqing Medical University with informed consent, and in accordance with the Declaration of Helsinki. Patient information is listed in Supplementary Table S2 and Table S3. Mononuclear cells were extracted from samples according to the Ficoll-Hypaque density centrifugation method. After extraction, erythrocytes were lysed and mixed, centrifuged again, washed with PBS, and cultured in 1640 medium plus 20% FBS, ITS Solution (Sigma-Aldrich, St Louis, MO, USA) and 20% supernatant of the 5637 bladder cancer cell line 17-19.

### Synergistic effect *in vitro* cell assay

The cells were seeded at a concentration of 0.2 × 10^5^ in 96-well plates and cultured at 37 °C with 5% CO2 and 95% humidity. The cells were treated with increasing concentration of gilteritinib and/or mitoxantrone-liposome. DMSO as used to treat cells in the blank control group. The Cell Counting Kit-8 (CCK-8) solution was added to each well 24 h post-treatment and the absorbance of the cells was measured at 450 nm using a multimode microplate reader (Molecular Devices, San Hose, CA, USA). The IC_50_ of the drug was obtained by calculating the cell survival rate at different drug concentrations using the following formula: Drug synergy was evaluated using a combination index (CI) equation based on the Chou-Talalay multiple-drug effect equation. The extent and direction of the anti-leukemic interaction between the two agents were determined by calculating the CI using CompuSyn software (ComboSyn Inc., Paramus, NJ, USA), where CI <1, CI =1, and CI >1 indicated synergistic, additive, and antagonistic effects, respectively 20,21.

### Real time-quantitative polymerase chain reaction (RT-qPCR)

Total RNA was extracted from the cells using TRIzol® reagent (Invitrogen, Carlsbad, CA, USA) according to the manufacturer's instructions. Complementary DNA (cDNA) was synthesized using a PrimeScriptTM RT Master Mix Kit (TaKaRa, Dalian, China). Real time PCR analysis was performed using SYBR® Premix Ex Taq (Tli RNase H Plus) (TaKaRa). Gene expression was normalized to that of the housekeeping control, GAPDH or β-actin. The amplification primers are shown in Supplementary Table S4.

### Cell proliferation, apoptosis, cell cycle, and flow cytometric analyses

Cell proliferation assays were performed using cell counter. The single-cell suspension was prepared by washing the cells with PBS, adding an appropriate amount of 1640 medium, and gently remixing the cells. The cell suspension, comprising approximately 10 μL, was aspirate the cell suspension and added to the counting plate, which was then placed in the automatic counter for counting for 1-2 minutes until the cells had settled. The apoptotic rate was confirmed by flow cytometry using an Annexin V-FITC/PI apoptosis detection kit (4A Biotech Co. Ltd., China). Cell cycle analysis was performed by flow cytometry using the Cell Cycle Analysis Kit (MultiSciences Biotech Co. Ltd., China).

### Western blot

The cells were lysed in the presence of protease and phosphatase inhibitors (Ovibion, China), and the whole-cell lysates were subjected to sodium dodecyl sulfate (SDS)-polyacrylamide gel electrophoresis. The proteins were then transferred to polyvinylidene difluoride membranes (0.45 μM, Merk Millipore Ltd., Tullagreen, Carrigtwohill, Co. Cork Ireland) and westen blot with antibodies against *FLT3* (ab245116; Abcam, Cambridge, MA, USA), AKT (ab8805; Abcam), p21 (AF6290), P-p21 (DF4570), CDK2 (AF6237), PI3K (tyr458), CyclinA (AF0142; Affinity Biosciences, Zhenjiang, Jiangsu, China), p53 (9286; Cell signaling Technology, Danvers, MA, USA), β-actin (66009-1-Ig; Proteintech, Rosemont, IL, USA). Western blotting was repeated at least three times, and a representative blot is shown.

### RNA-sequencing (RNA-seq) analysis

Total RNA was isolated from the single/combination drug and control MV4-11 cells using the TRIzol® Reagent. RNA-seq analysis was performed at Shanghai Tissuebank Diagnostics Co. Ltd. (China) (Supplementary information).

### AML xenograft model and treatment

NOD. Cg-Prkdcscid IL2 rgtm1Wjl/ SzJ (NSG) mice (weighing 20 g) were purchased from Vital River (Beijing, China) and maintained at the animal experimental center of the Chongqing Medical University. For *in vivo* experiments, the mice were injected via the caudal vein with 1 × 10^7^ MV4-11 cells. After seven days, the animals were randomized into the following treatment groups: vehicle (DMSO), mitoxantrone (5 mg/kg, day 7 injection), and mitoxantrone-liposome (5 mg/kg, day 7 injection). After day 21, the mice were euthanized and the leukemia burden, spleen size, and body weight were assessed. During treatment, the drug concentration in the BM and peripheral blood of the AML xenograft model mice were measured at 24 h, 72 h, and 1 week (Supplementary Figure S1). In another experiment, animals were randomized into four groups: vehicle (DMSO), mitoxantrone-liposome (5 mg/kg, day 1 injection), gilteritinib (30 mg/kg, 28 consecutive days, orally), and a combination of both drugs. After treatment, the leukemic burden, spleen size, and body weights of the mice were assessed. For analysis of survival, treatment was initiated on day 7 post-transplantation in the same manner as in the previous ah, and observation continued until day 120. All mouse experiments were approved by the Animal Ethics Association of the Chongqing Medical University.

### Statistical analysis

Statistical analyses were performed as indicated using R language (version 3.5.2) or GraphPad Prism (version 8.0.0). Two-sample t-tests were used to compare two groups. Survival curves were constructed using the Kaplan-Meier method. Survival data were evaluated by univariate and multivariate Cox regression analyses. Bioinformatic approaches of Gene Ontology (GO) analysis, Kyoto Encyclopedia of Genes and Genomes (KEGG) analysis, and Gene set enrichment analysis (GSEA) were performed using the OmicStudio tools (https://www.omicstudio.cn/tool). P < 0.05 was considered statistically significant.

## Results

### Mitoxantrone-liposomes efficiently inhibit AML cells, particularly *FLT3-ITD* mutated AML cells, *in vivo* and *in vitro*

**Figure 1:**
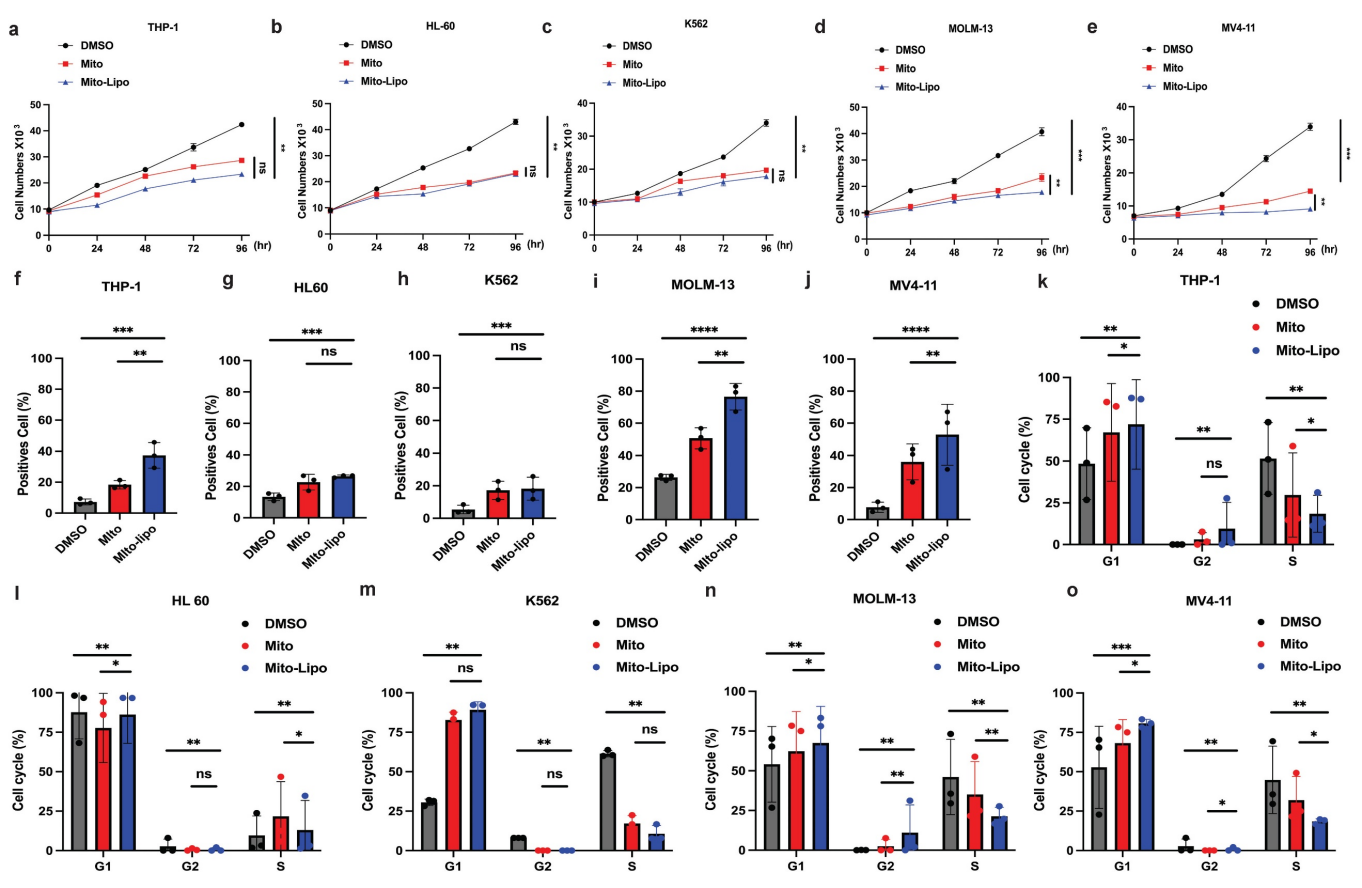
** Mitoxantrone-liposomes efficiently inhibit AML cells, particularly *FLT3-ITD* mutated AML cells, *in vivo* and *in vitro*. a-e.** Cell proliferation was measured at different time points (0, 24, 48, 72, 96h) in THP-1, HL-60, K562, MV4-11, MOLM13 cells after treatment (mitoxantrone-liposome 20 nM and mitoxantrone treatment 20 nM). **f-j.** Flow cytometry (representative images are presented in Fig. S1-i) was used to confirm the apoptosis by treatment in THP-1, HL-60, K562, MOLM13, MV4-11 (mitoxantrone-liposome 20 nM and mitoxantrone treatment 20 nM). k-o. Flow cytometry (representative images are presented in Fig. S1-j) was used to analyze the cell cycle in THP-1, HL-60, K562, MOLM13, MV4-11 (mitoxantrone-liposome 20 nM and mitoxantrone treatment 20 nM). (Mito: mitoxantrone, Mito-Lipo: mitoxantrone-liposome).

**Figure 2:**
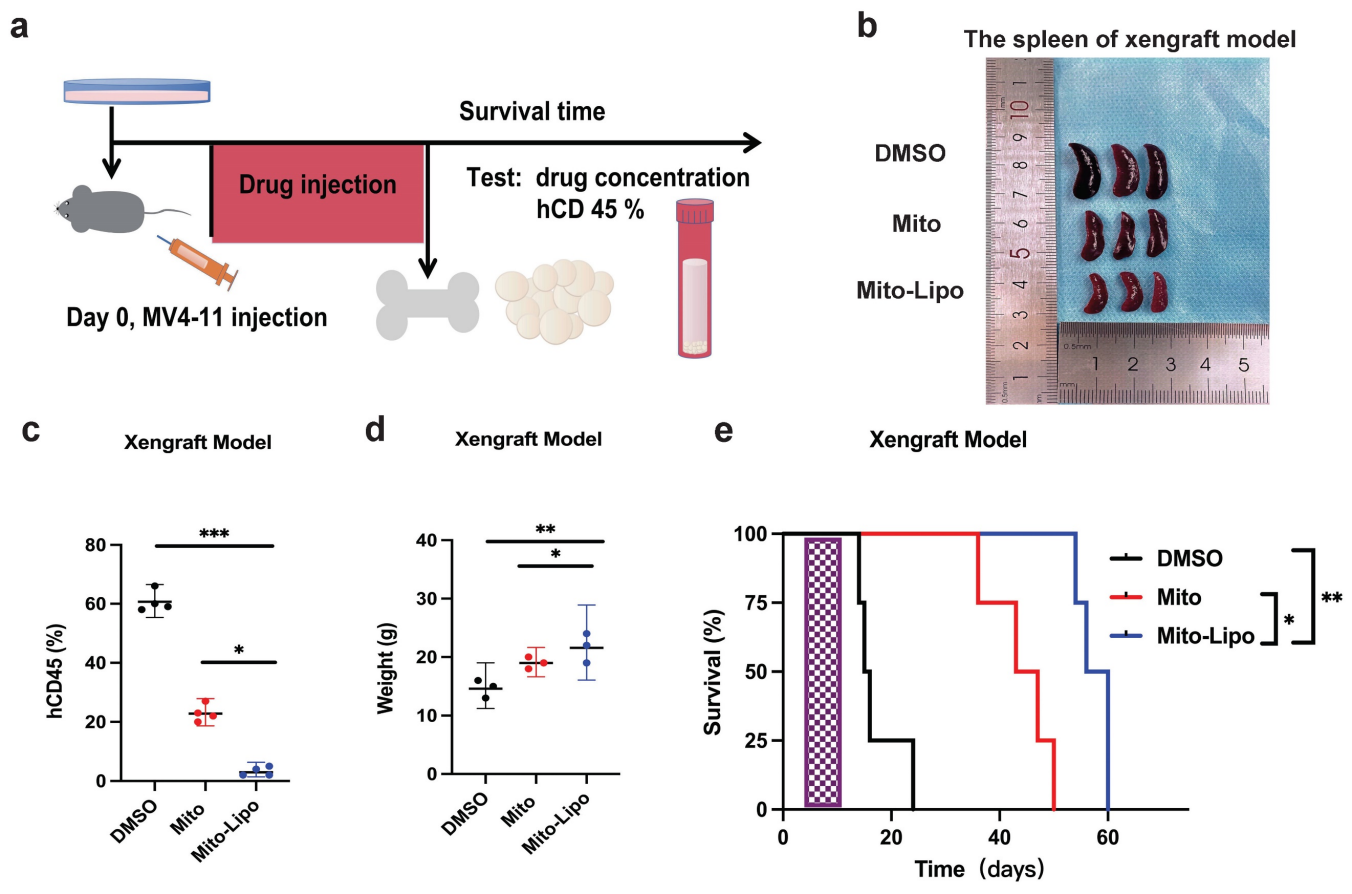
** Mitoxantrone compared the efficacy of mitoxantrone-liposome *in vivo*. a.** Flow chart of experimental therapy in AML xenograft model. **b.** Experimental set up for the treatment of MV4-11 xenograft mice, percentage of hCD45 cells in the BM of leukemic mice after treatment with DMSO, Mitoxantrone (5 mg/kg, Day 7 for injection), mitoxantrone-liposome (5 mg/kg Day 7 for injection). **c.** The sizes of spleens were measured at the end of the study. **d.** The body weights of the MV4-11 xenograft leukemic xenograft mice were measured before euthanasia. **e.** Kaplan-Meier survival analysis of MV4-11 xenograft mice treated with DMSO, mitoxantrone (5 mg/kg, Day 7 for injection), mitoxantrone-liposome (5 mg/kg, Day 7 for injection). The log-rank (Mantel-Cox) test was used to calculate the P-values. *P < 0.05; **P < 0.01; ***P < 0.001. (Mito: Mitoxantrone, Mito-Lipo: Mitoxantrone-liposome).

We first compared the therapeutic effects of mitoxantrone-liposomes and mitoxantrone on leukemia cell lines Thp-1, K562, HL-60, MV4-11, and MOLM13. Cell proliferation assay revealed that mitoxantrone-liposomes exhibited a significantly higher therapeutic effect in *FLT3-ITD* AML cell lines than mitoxantrone (Fig. 1a-e). Compared to mitoxantrone alone, flow cytometry analysis of apoptosis and the cell cycle after 24 h of treatment revealed a markedly greater effect in MV4-11 and MOLM13 cells (Fig. 1f-j, Fig. 1k-o and Fig. S1i-j). AML xenograft mouse models were established and randomly divided into two groups (n = 15 per group). Seven days after model generation, the mice were injected with mitoxantrone-liposomes or mitoxantrone. Mitoxantrone concentrations in the bone marrow (BM) and peripheral blood of the two groups were measured at 24 h, 72 h, and 1 week. The results showed that the concentration of mitoxantrone-liposomes in the BM and peripheral blood was higher than that of mitoxantrone (Fig. S1a-h). In a separate experiment, AML xenograft mouse models were established, and the mice were randomly divided into three groups (n = 5 per group) (Fig. 2a). The body weight of mice in the mitoxantrone-liposomes treatment group was higher than that of mice in the mitoxantrone group (Fig. 2d, Fig. S6). Herein, the expression of human CD45 (hCD45) in the BM of AML xenograft model mice was used as an indicator of the leukemic burden. The hCD45 levels in AML xenograft model mice were sacrificed 14 d post-treatment were assessed using flow cytometry (Fig. 2c). The results indicated an obvious anti-leukemic effect of mitoxantrone-liposomes compared to mitoxantrone. Spleen size in the mitoxantrone-liposomes-treated group was smaller than that in the mitoxantrone-treated group (Fig. 2b). In another experiment, the AML xenograft mouse model was established, the mice were randomly divided into three groups (n = 5 per group), and the survival of mice in the three treatment groups was assessed (Fig. 2e). The results revealed that mitoxantrone-liposomes had a significantly stronger antileukemic effect, especially in *FLT3-ITD* AML cell lines, and showed better efficacy and safety than mitoxantrone.

### Combination of mitoxantrone-liposomes and gilteritinib exert synergistic effect against* FLT3-ITD* AML cell lines *in vitro*

**Figure 3:**
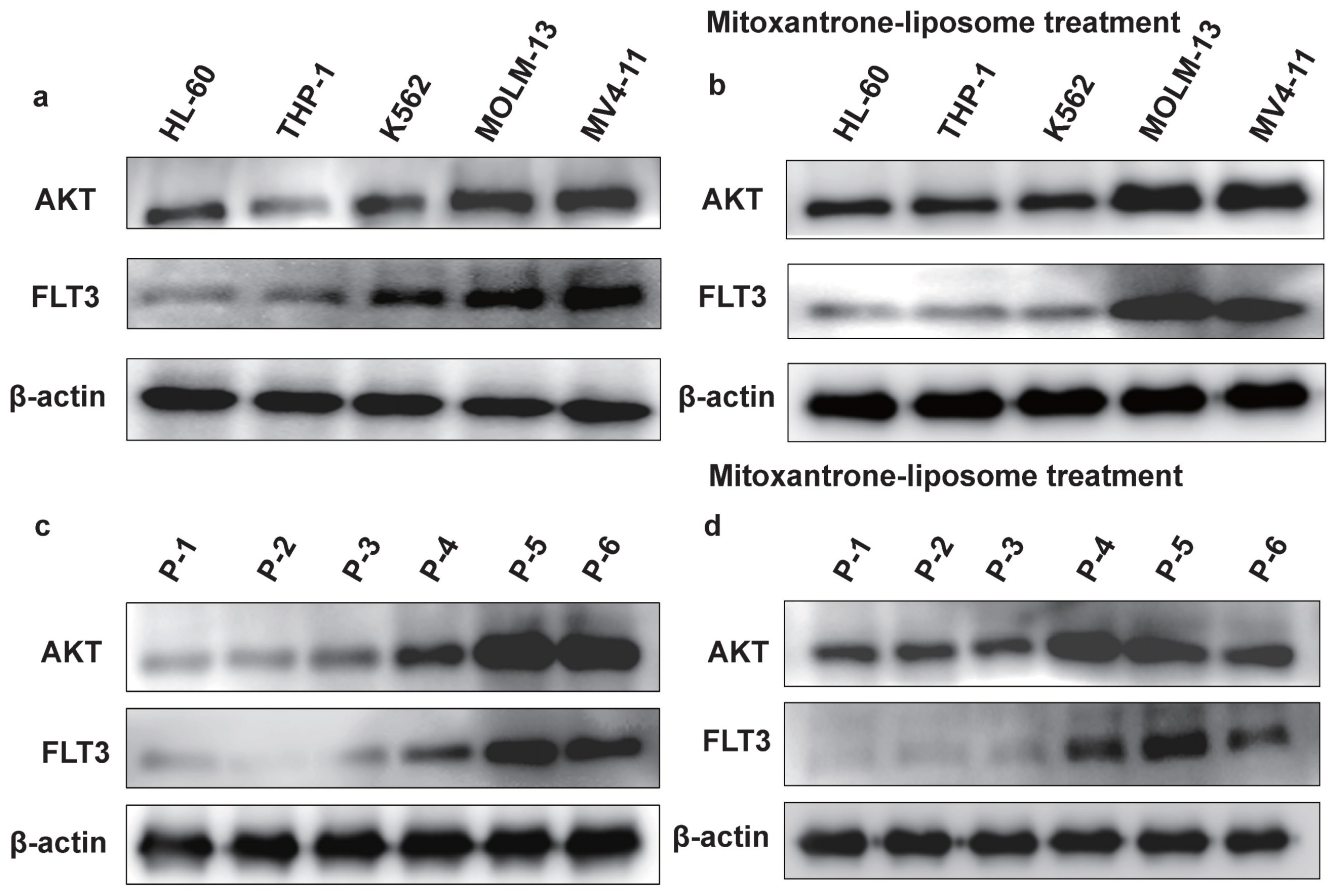
** The protein expression level of FLT3 and AKT in cell lines. a.** The protein expression level of FLT3 and AKT in HL-60, THP-1, K562, MOLM13, MV4-11 leukemia cell lines were measured by western blotting before mitoxantrone-liposome (20 nM) treatment. **b.** The protein expression level of FLT3 and AKT in HL-60, THP-1, K562, MOLM13, MV4-11 leukemia cell lines were measured by western blotting after Mitoxantrone-liposome (20 nM) treatment. **c.** The protein expression level of FLT3 and AKT in leukemia patient were measured by western blotting before Mitoxantrone-liposome (20 nM) treatment (P1-P3: AML patient, P5-P6: *FLT3-ITD* AML patient). **d.** The protein expression level of FLT3 and AKT in leukemia patient were measured by western blotting after mitoxantrone-liposome (20 nM) treatment (P1-P3: AML patient, P5-P6: *FLT3-ITD* AML patient).

**Figure 4:**
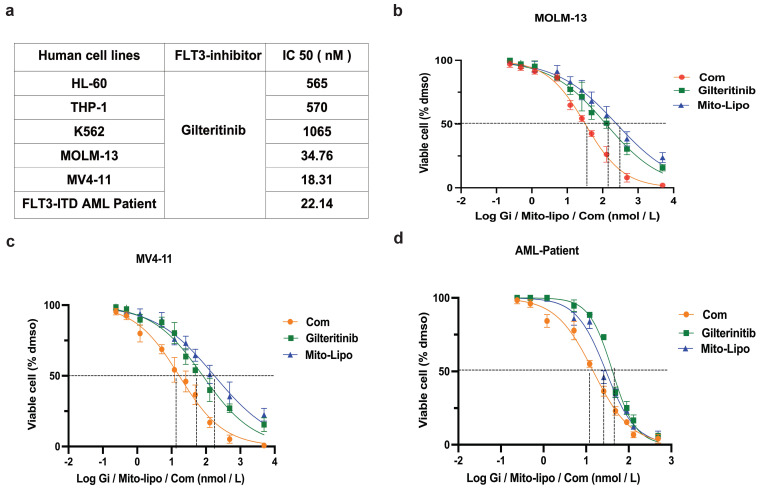
** Combination of mitoxantrone-liposomes and gilteritinib in *FLT3-ITD* AML cell lines *in vitro*. a.** Summary of FLT3 inhibitor IC_50_ concentration in HL-60, THP-1, K562, MOLM13, MV4-11 leukemia cell lines and *FLT3-ITD* AML patient cells.** b-d.** Dose-response curves from cell-viability assays after 24 hours of treatment with mitxantrone-liposome, Gilteritinib, Combination in MOLM13, MV4-11 and *FLT3-ITD* AML leukemia patients, IC_50_ values were graphically determined by Graphpad prism. (Com mitoxantrone-liposome and giltertinib; Mito-Lipo: mitoxantrone-liposome).

**Figure 5:**
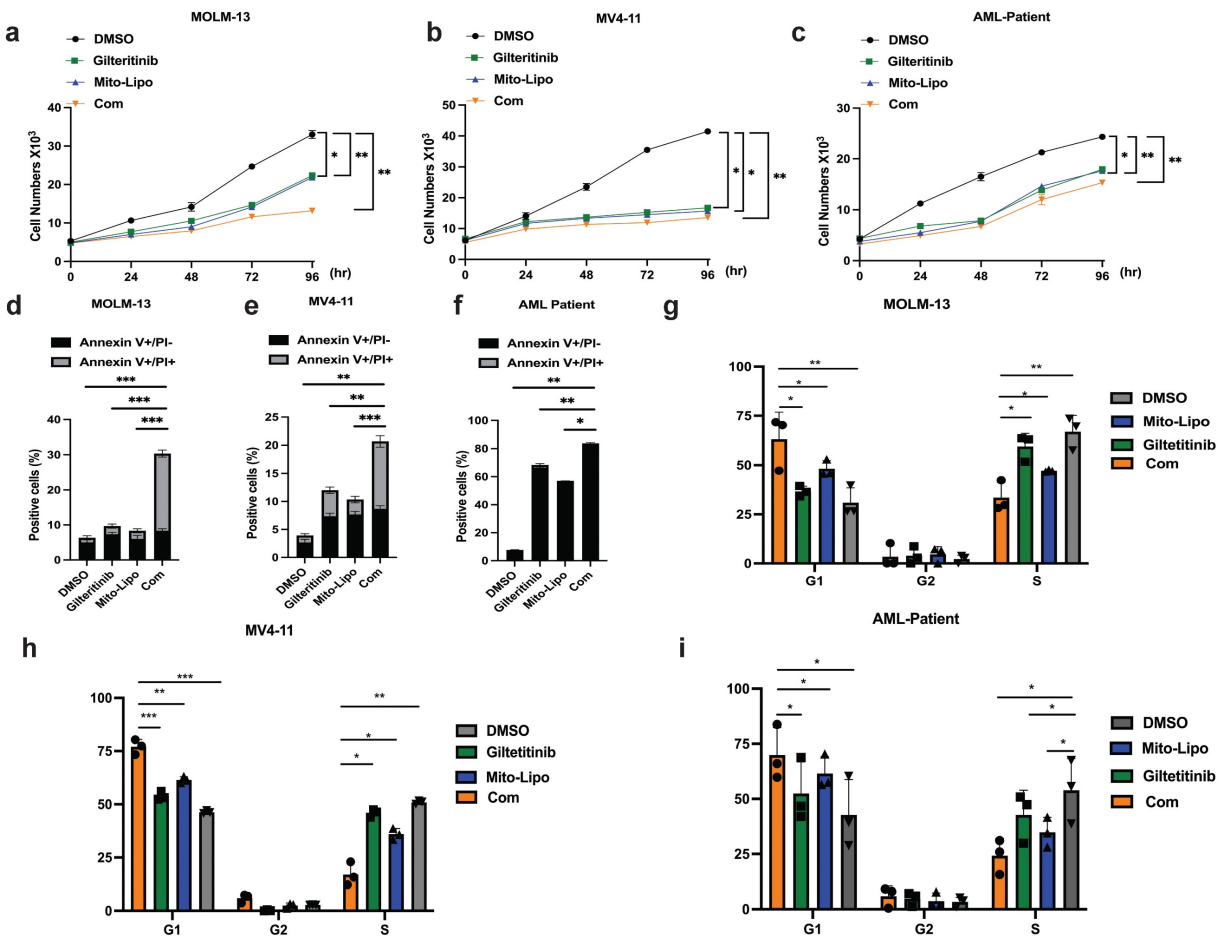
** Combination of mitoxantrone-liposomes and gilteritinib exert synergistic effect against *FLT3-ITD* AML cell lines *in vitro*. a-c.** Cell proliferation was measured at different time points (0, 24, 48, 72, 96h) in MV4-11, MOLM13 and *FLT3-ITD* AML Patient cells after treatment (mitoxantrone-liposome 20 nM and mitoxantrone treatment 20 nM). d-f. Flow cytometry (representative images are presented in supplementary) was used to confirm the apoptosis (Annexin V/PI- and Annexin V/PI+) in MOLM13, MV4-11, *FLT3-ITD* AML patient cells after 24h treatment (gilteritinib: 25 nM, mitoxantrone-liposome: 20 nM, concentrations of combination durg: gilteritinib 20 nM, mitoxantrone-liposome 20 nM). j-i. Flow cytometry (representative images are presented in supplementary) was used to analyze the cell cycle in MOLM13, MV4-11, *FLT3-ITD* AML patient cells after 24h treatment (gilteritinib: 25 nM, mitoxantrone-liposome: 20 nM, combination drug: gilteritinib 20 nM, mitoxantrone-liposome 20 nM). Error bars represent means ± SD. *, P < 0.05, **, P < 0.01, ***, P < 0.001; n.s. no significance; Student's t test or one-way ANOVA. (Com: Combination: mitoxantrone-liposome and giltertinib; Mito-Lipo: mitoxantrone-liposome).

**Figure 6:**
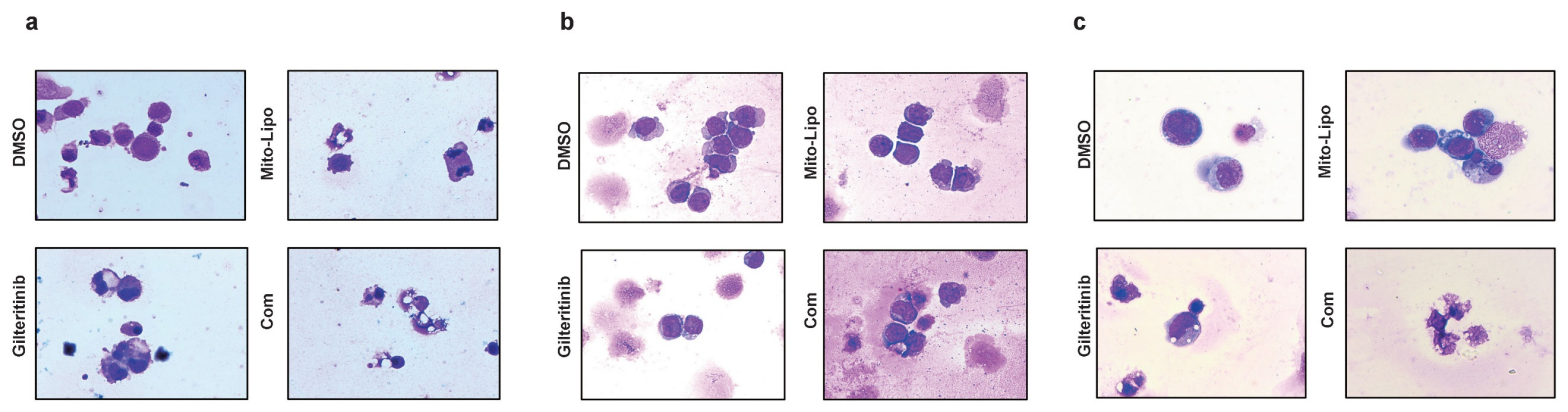
** Cell morphological changes after treatment. a-c.** Swiss staining showing MV4-11 and MOLM13 and *FLT3-ITD* AML patient cells after Mitoxantrone-limposome or Gilteritinib or Combination and DMSO. Micrographs were taken at 100X amplification, error bars reprent SD of 3 independent experiments, each performed in 3 technical replicates. (Com: Combination: mitoxantrone-liposome and giltertinib).

We examined the effect of combination treatment on *FLT3* wild-type AML (THP-1, HL-60, and K562) and *FLT3-ITD* mutant AML cell lines (MOLM13 and MV4-11). We first determined the FLT3 and AKT protein levels in luekemia cell lines before mitoxantrone-liposomes treatment (Fig. 3a), then determined the FLT3 protein levels in the leukemia cell lines 12 h post-treatment with mitoxantrone-liposomes (Fig. 3b). Consequently, the results showed lower expression levels of the protein in THP-1, HL-60, and K562 cells compared to that in MOLM13 and MV4-11 cells (Fig. 3a-b). We first verified the effect of gilteritinib on THP-1, HL-60, K562, MOLM13, MV4-11, and *FLT3*-ITD AML Patient cells (Fig. 4a). MOLM13 and MV4-11 *FLT 3-ITD* mutant cell lines were used in this study. MOLM13 and MV4-11 showed greater synergistic effects than THP-1, K562, and HL-60 cells (Table S1). Drug Dose and Effect Curve Results Indicated Superiority of Combination Therapy Over Monotherapy (Fig. 4b-c). CCK8 was evaluated to assess the proliferation of MOLM13, MV4-11 after combination therapy and monotherapy, and the results suggested that the combination therapy led to a decrease in the proliferation of tumor cells (Fig. 5a-b). Based on the synergistic effect of combination treatment, we assessed annexin V-positive cells and cell cycle in MV4-11 and MOLM13 cells (Fig. 5d-f, Fig. 5g-h, Fig. S2, Fig. S3), using flow cytometry, after 24 h of combined treatment compared to treatment with single drugs or dimethylsulfoxide (DMSO). The results suggested that combined treatment increased apoptosis and decreased the S phase. Morphological changes in *FLT3-ITD* AML cells were identified using Swiss staining (Fig. 6a-b). Thus, combination therapy with mitoxantrone-liposomes and gilteritinib promoted apoptosis of leukemia cells and reduced DNA replication in the S phase.

### Combined treatment with gilteritinib and mitoxantrone-liposomes suppress primary *Flt3-ITD* AML cells *in vitro*

In the present study, we collected BM samples from patients with *FLT3-ITD* AML and extracted mononuclear cells for further experiments. First, we compared AKT protein expression in samples from patients with *FLT3-ITD* AML pre- and post-treatment with mitoxantrone-liposomes, and found increased expression of the AKT protein post-treatment (Fig. 3c-d). We then determined the half-maximal inhibitory concentration of the specific and potent FLT3 inhibitor, gilteritinib, in human *FLT3-ITD* leukemia cell lines after 24 h of treatment (Fig. 4a). Drug Dose and Effect Curve Results Indicated Superiority of Combination Therapy Over Monotherapy (Fig. 4d). Proliferation was measured after drug treatment for 24 h (Fig. 5c). Flow cytometry was performed to detect apoptosis and cell cycle progression, which showed that apoptosis was significantly increased in these cells compared to cells treated with monotherapy and DMSO (Fig. 5f), and the S phase was significantly shortened (Fig. 5i). These data indicate that combination treatment increased apoptosis and decreased the S phase compared to single-drug treatment in cells from patients with *FLT3-ITD* AML. Morphological changes in these cells were identified using Swiss staining (Fig. 6c), and the underlying mechanism was verified using RNA-seq. Patient information is shown in supplementary (Table S2, Table S3).

### Gilteritinib and mitoxantrone-liposomes synergistically activate p53 pathway, enhance p21 phosphorylation, and decrease cyclin A/CDK2 expression

**Figure 7:**
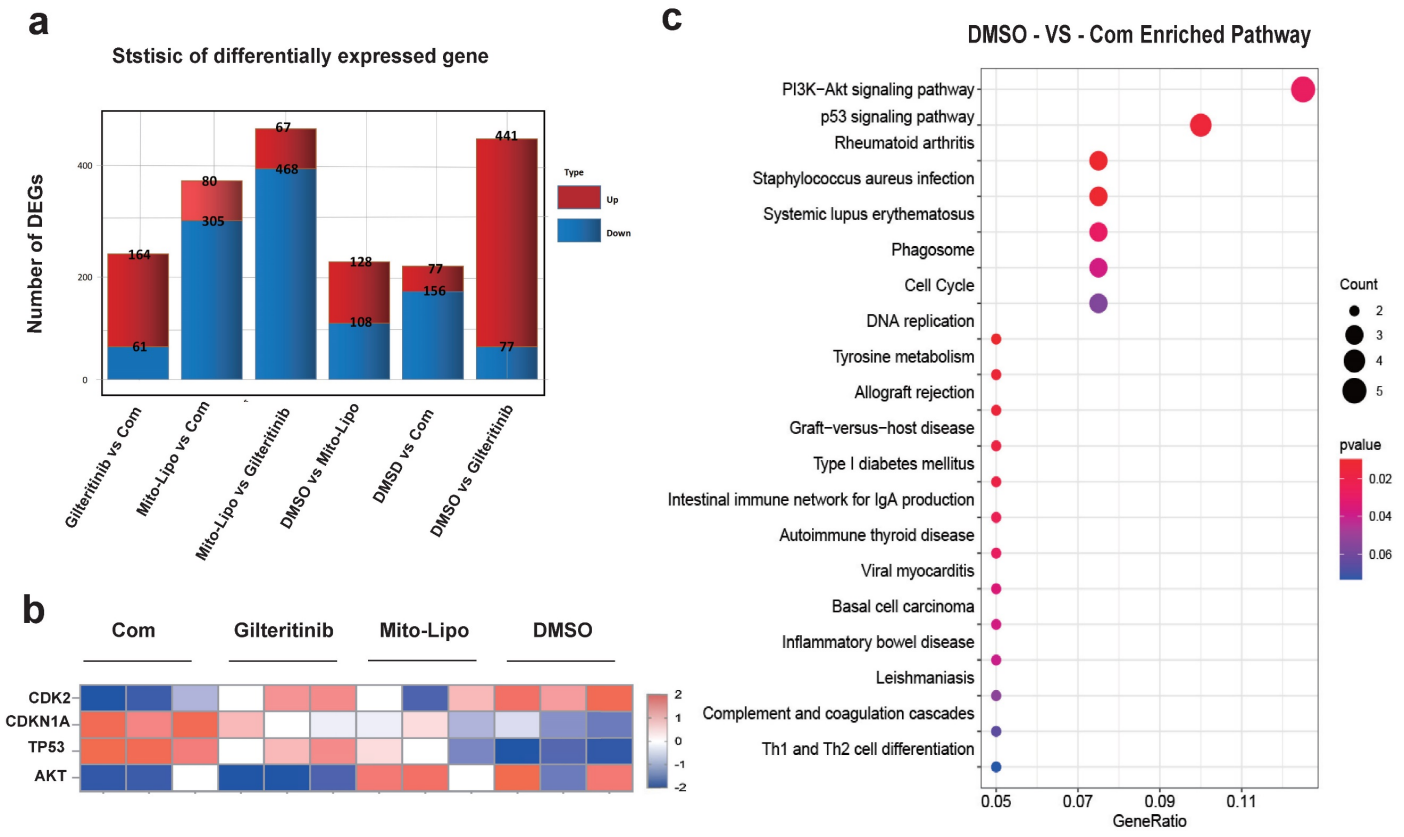
** Gene expression. a.** Differentially expressed gene, MV4-11 cells were treated with Gilteritinib, Mitoxantrone-liposome, Combonation and DMSO, Cells were collected at 24 hours for the transcriptome analysis, in each condition, the number of differentially expressed genes (averaged for four samples) with false discovery rate (FDR) values < 0.05 is shown (Gilteritinib: 25 nM, mitoxantrone-liposome: 20 nM, concentrations of combination drug: gilteritinib 20 nM, mitoxantrone-liposome 20 nM). **b.** Heat map representation of average expression of CDK2, CDKN1A, TP53, AKT for the four samples in **a** for each treatment condition. **c.** Kyoto Encyclopedia of Genes and Genomes (KEGG) analyses revealed the potential roles of signal pathway. Error bars represent means ± SD. *, P < 0.05, **, P < 0.01, ***, P < 0.001; ns. no significance; Student's t test or one-way ANOVA. (Com: Combination: Mitoxantrone-liposome and giltertinib, Mito-Lipo: mitoxantrone-liposome).

The combination of gilteritinib and mitoxantrone-liposomes produced a significant synergistic effect on *FLT3-ITD* AML cells (Table S1). To examine the mechanism underlying the synergistic effects of the combination therapy, we performed RNA-seq analysis in MV4-11 cells and compared the changes in the combination treatment with those of single drug or DMSO treatment. Gene set enrichment analysis showed that the genes changes between drug treatments (Fig. 7a-b), and that the PI3K-AKT / P53 / Cell cycle may be the mechanism of the synergistic therapeutic effect by the Kyoto encyclopedia of genes and genomes analysis (Fig. 7c, Fig. S8).

**Figure 8:**
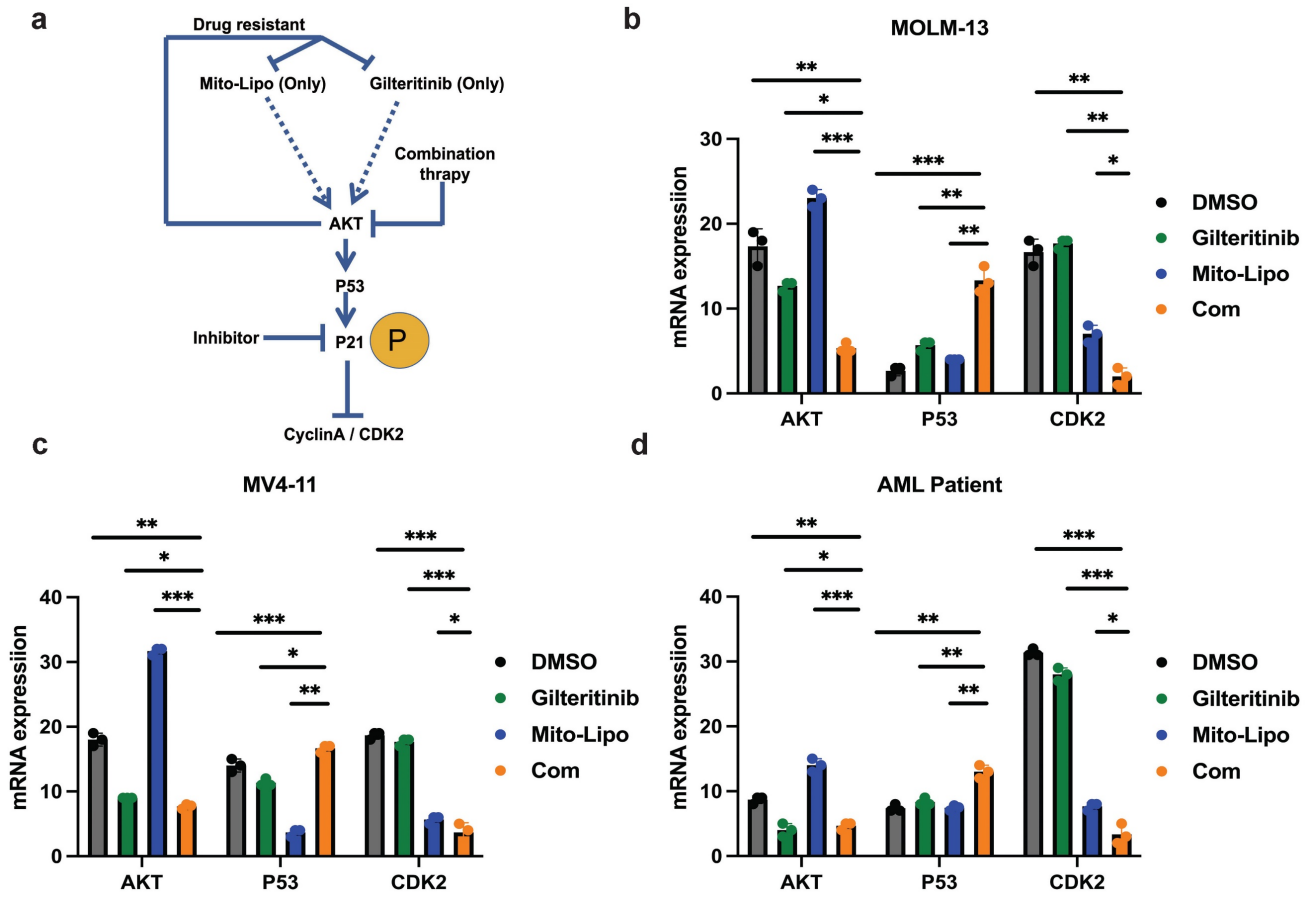
**Effects of single and combined mitoxantrone-liposome and gilteritinib on relative protein levels. a.** Diagram of the mechanism of treatment. **b-d.** The mRNA expression levels of P53, AKT and CDK2 were measured by real-time qpcr after 12 h treatment in MOLM13, MV4-11 and *FLT3-ITD* AML Patient cells. Error bars represent means ± SD. *, P < 0.05, **, P < 0.01, ***, P < 0.001; n.s. no significance; Student's t test or one-way ANOVA. (Com: Combination: mitoxantrone-liposome and giltertinib; Mito-Lipo: mitoxantrone-liposome).

**Figure 9:**
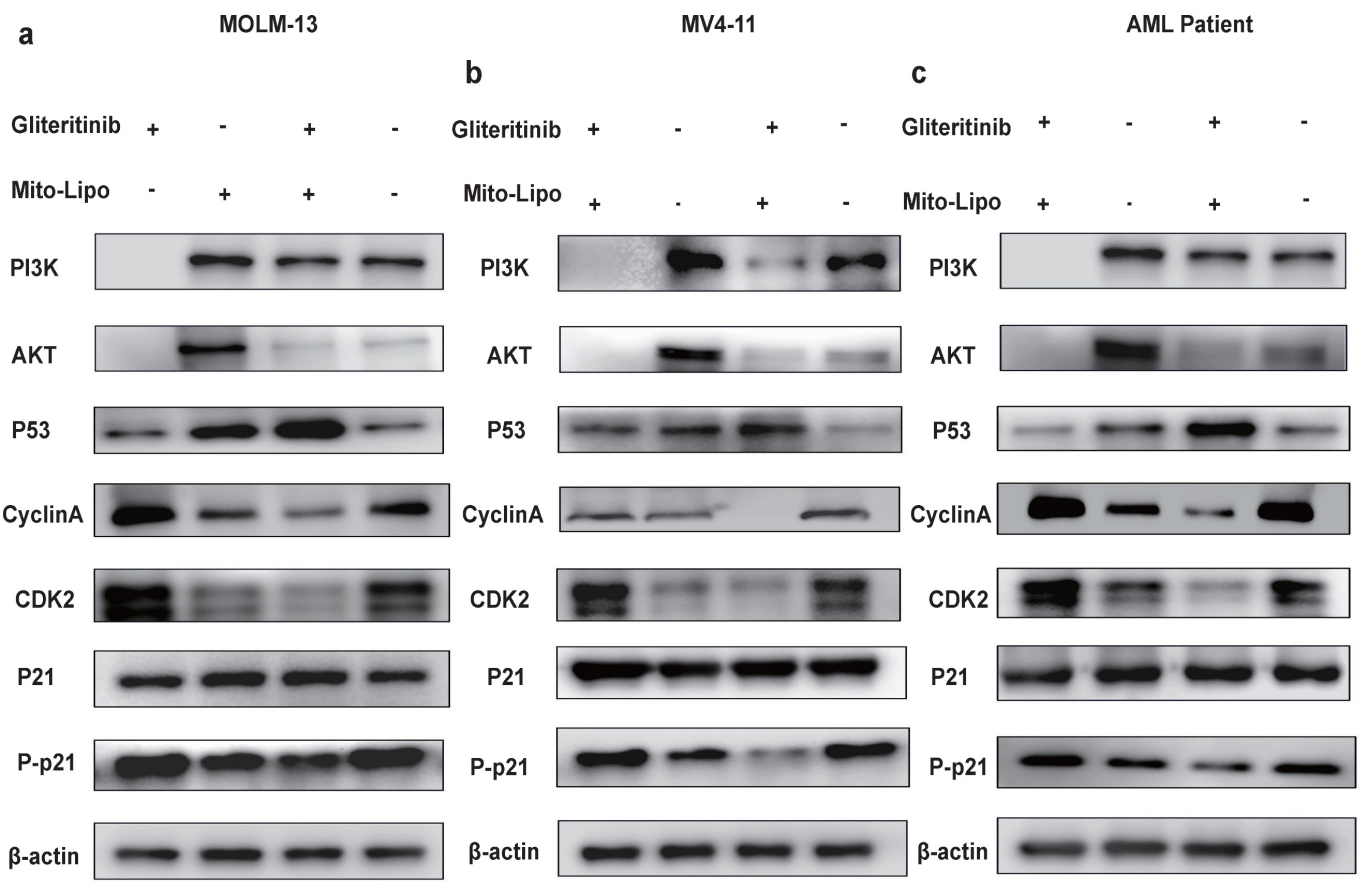
** a-c.** The protein expression levels of PI3K, AKT, P21, Phosphorylation P21, CyclinA/CDK2 were measured by western blotting after 12 h treatment in MOLM13, MV4-11 and *FLT3-ITD* AML patient cells. Error bars represent means ± SD. *, P < 0.05, **, P < 0.01, ***, P < 0.001; n.s. no significance; Student's t test or one-way ANOVA. (Mito-Lipo: mitoxantrone-liposome, AML Patient: *FLT3-ITD* AML patient).

**Figure 10:**
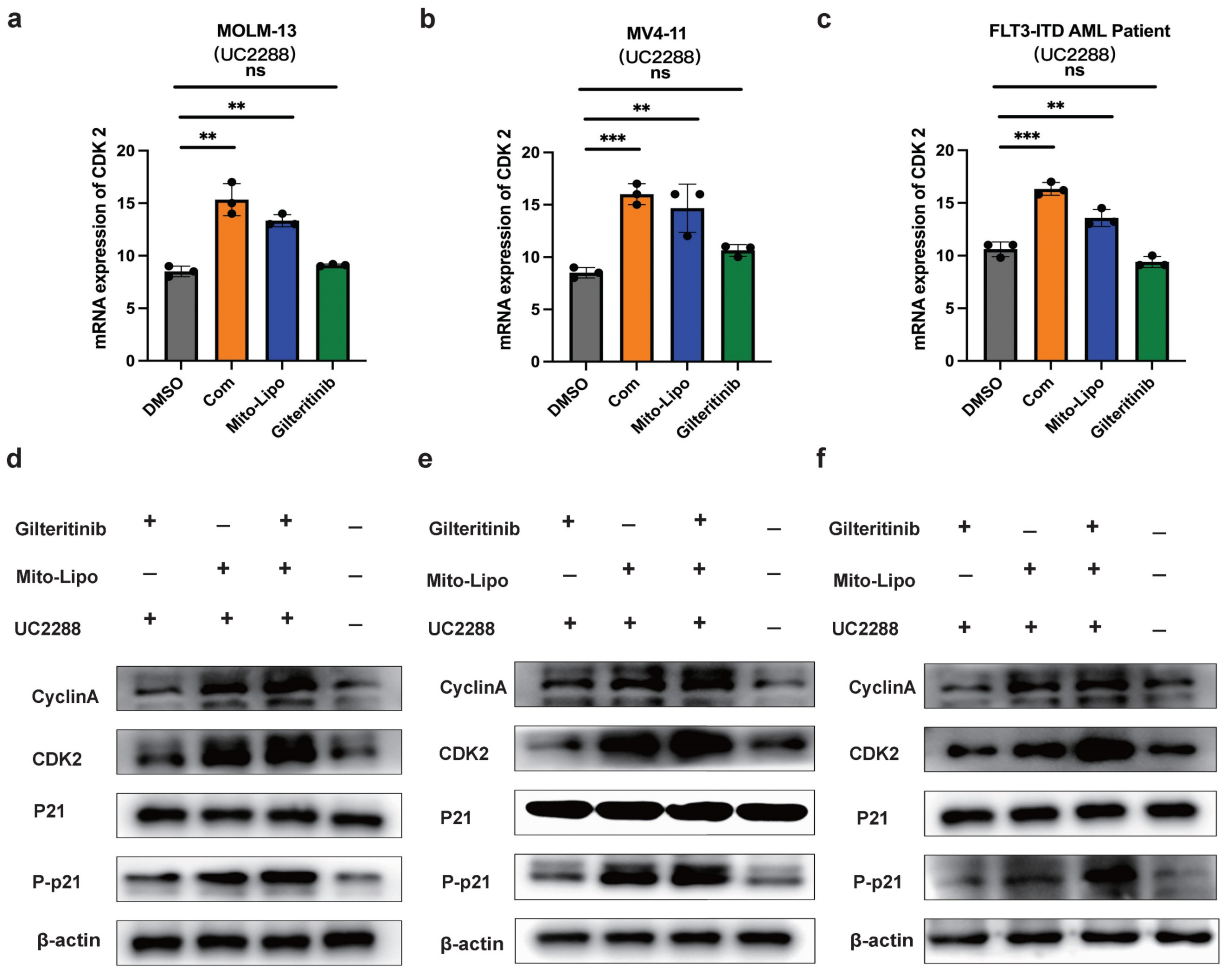
** a-c.** The mRNA expression levels of CDK2 were measured by real-time qpcr after using drug and P21 inhibitor (UC2288) after 12 h treatment in MOLM13, MV4-11 and* FLT3-ITD* AML patient. d**-f.** The protein expression level of P21, Phosphorylation P21, CyclinA/CDK2 were measured by western blotting after using drug and P21 inhibitor (UC2288) after 12 h in MOLM13, MV4-11 and *FLT3-ITD* AML patient cells. Drug concentrations: Gilteritinib 25 nM, Mitoxantrone-liposome 20 nM, Combination: gilteritinib: 20 nM, mitoxantrone-liposome: 20 nM, P21 inhibitor (UC2288): 10 nM. Error bars represent means ± SD. *, P < 0.05, **, P < 0.01, ***, P < 0.001; n.s. no significance; Student's t test or one-way ANOVA.

The combination treatment may activate the p53 pathway, increased p21 phosphorylation, and decreased AKT levels (Fig. 8a). To further examine the mechanism underlying the combination therapeutic effect, we evaluated the mRNA expression of p53 and AKT following combination treatment and found that the expression of p53 increased, whereas that of AKT decreased (Fig. 8b-d). Western blotting was used to evaluate the p53 protein levels in the monotherapy and combination therapy groups (Fig. 9a-c). P53 protein levels were significantly higher in the combination therapy group than in the monotherapy group, indicating that combination therapy enhanced the activation of the p53 pathway. Simultaneously, we analyzed changes in the level of P-p21 (Thr145) protein, which is downstream of p53, and found that its level was significantly lower in the combination drug group than that in the other groups (Fig. 9a-c, Fig. S4). Subsequently, using the p21 inhibitor (UC2288), we evaluated whether targeting the p53 pathway or CDKs alters the mRNA expression of *CDK2* (Fig. 10a-c) and the cyclin A/CDK2 and P-p21 protein expression in *FLT3-ITD* AML (Fig. 10d-f, Fig. S5). The results showed that the protein levels of P-p21 (Thr145) and cyclin A/CDK2 increased in *FLT3-ITD* AML cells. These data demonstrate that a combination of mechanisms occur in the treated cells (Fig. 8a).

### Mitoxantrone-liposomes and gilteritinib synergistically exert powerful anti-leukemic activity in an *FLT3*-ITD AML xenograft model

**Figure 11:**
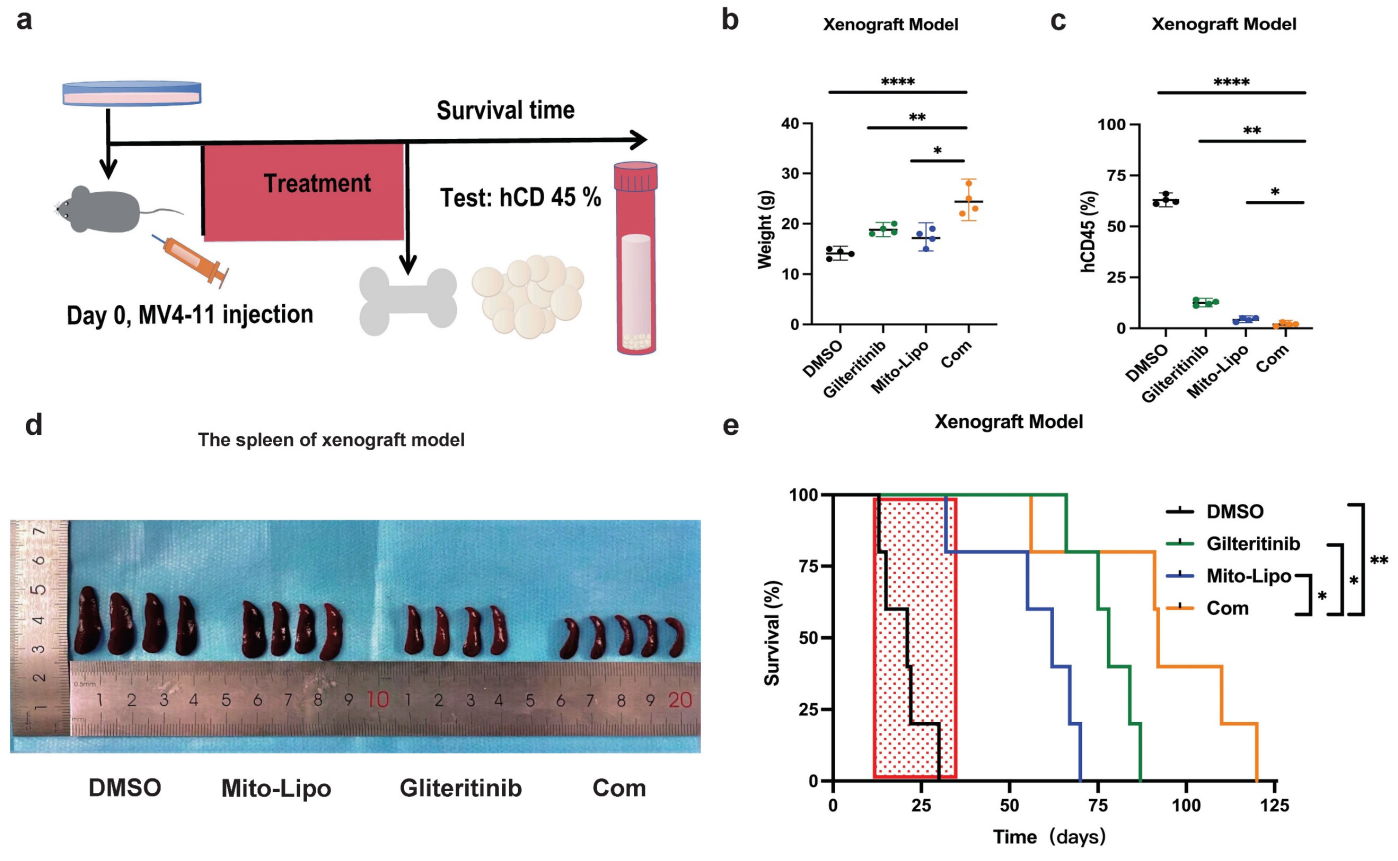
** Effects of treatment in AML xenograft model. a.** The chart of experimental therapy in AML xenograft model. **b.** The sizes of spleens were measured at the end of the study. **c.** The weight of the MV4-11 xenograft mice. **d.** Experimental set up for the treatment of MV4-11 xenograft mice (n=5), percentage of human CD45 cells in the bone marrow of MV4-11 xenograft mice after treatment. **e.** Kaplan-Meier survival analysis of MV4-11 xenograft mice treated with DMSO, Mitoxantrone-liposome (5 mg/kg, day 7 for injection), Giltetitinib (30 mg/kg, 28 consecutive days, Per os) and Combination. The log-rank (Mantel-Cox) test was used to calculate the P-values. *P < 0.05; **P < 0.01; ***P < 0.001. (Com: Combination: mitoxantrone-liposome and giltertinib; Mito-Lipo: mitoxantrone-liposome).

Furthermore, we examined the therapeutic potential of mitoxantrone-liposomes in combination with gilteritinib *in vivo*. First, we assessed the effect of these drugs on leukemic burden in an *FLT3-ITD* AML xenograft model. MV4-11 cells were transplanted into NSG mice via tail vein injection, and the animals were randomly divided into four groups (n = 5) that received mitoxantrone-liposomes, gilteritinib, combination, or DMSO treatment (Fig. 11a). Treatment was started 7 days after transplantation, and the animals were euthanized on the 36^th^ day.

Assessment of hCD45 expression in the BM of mice from the four groups revealed the following expression pattern: DMSO, 60.44%; mitoxantrone-liposomes, 12.20%; gilteritinib, 8.254%; combination, 2.345%; p <0.05 for combination vs other groups) (Fig. 11c). We also measured spleen size (Fig. 11c) and recorded body weights (Fig. 11b, Fig. S7). Then, we assessed the survival time of the *FLT3-ITD* AML xenograft model mice until 120 d post-treatment. The results showed that combination treatment significantly prolonged the survival time of the *FLT3-ITD* AML xenograft model mice (hazard ratios for death and 95% confidence intervals: combination vs gilteritinib, 0.466-4.132 and 0.242 - 2.142; combination vs mitoxantrone-liposome, 0.5698 - 6.649 and 0.1504 - 1.755; combination vs DMSO, 0.9884 - 9.813 and 0.1019 - 1.012) (Fig. 11e).

In summary, our data demonstrate that the combination of gilteritinib and mitoxantrone-liposomes substantially improves the survival of *FLT3-ITD* AML xenograft model mice compared to a single-drug or DMSO treatment and supports the clinical application of this therapeutic option as a novel approach for leukemia treatment.

## Discussion

Intensive chemotherapy remains the primary treatment for AML. However, relapse after chemotherapy remains a common problem 22. Currently, *FLT3* mutations account for a large proportion of both early and late AML diagnoses and relapsed or refractory AML 23-26. FLT3 inhibitors have been shown to increase the survival rates in de novo and in relapsed or refractory AML 23-26. However, emerging resistance mutations have been reported as a reason for FLT3 inhibition resistance; *FLT3* mutations do not exist in the AML primary clone, thus, they are not harmed by the FLT3 inhibitors 25,26. Due to its heterogeneity, single-drug treatment is ineffective in treating *FLT3-ITD* AML 27. In the past, *FLT3-ITD* AML treatment was primarily based on the "3+7" model, and the recurrence rate was higher 22.

In this study, we discovered that mitoxantrone-liposome and gilteritinib synergistically eradicated *FLT3-ITD* AML cells both *in vivo* and *in vitro*. A previous study reported that mitoxantrone may activate the* AKT* pathway in tumor cells during treatment, promoting drug resistance in tumor cells, thereby affecting the positive outcome of this treatment 28,29. However, in this study, RNA-seq results revealed that the combination drugs treatment inhibited AKT activation due to the therapeutic effects of gilteritinib 30,31. Simultaneously, RNA-seq revealed that combination treatment also inhibited early resistance to gilteritinib 32. These findings are interesting and warrant further research. Several studies are currently focused on the combination treatment with FLT3-inhibitor in *FLT3-ITD* AML; metformin exhibits a striking synergistic effect with gilteritinib in treating *FLT3-ITD* AML 33. The combination of FLT3 inhibitors with venetoclax is effective *in vitro* and* in vivo* against multiple models of FLT3-ITD-driven AML 34. Azacitidine plus venetoclax is the standard of care for patients with newly diagnosed AML who are unfit for intensive chemotherapy 34,35, and the study is currently in the experimental clinical phase II. Therefore, our study on mitoxantrone-liposomes combined with gilteritinib provides a new therapeutic strategy for treating *FLT3-ITD* AML.

In this study, anthracyclines reduce the side effects of cardiotoxicity by packaging liposomes. The side effects of mitoxantrone-liposomes were demonstrated *in vivo* by comparing the body weights and survival rates of mice post-drug treatment. This study may also suggest benefits from this new treatment regimen for older patients with AML and *FLT3-ITD* mutations. However, this study has some limitations. The effect time of mitoxantrone-liposomes in mice was longer than that of the drug without liposomes. Therefore, the change in the drug's half-life period when applied *in vivo* is closely related to the dose, and it is necessary to conduct *in vivo* or clinical experiments to find a suitable drug dose and administration frequency for patients with *FLT3-ITD* / *FLT3-ITD* relapsed or refractory AML. We are currently enrolling older patients with AML for this clinical study, and the results will be reported upon study completion.

In summary, our data suggest that mitoxantrone-liposome sensitizes *FLT3-ITD* AML cell lines and patients with *FLT3-ITD* AML primary samples to gilteritinib. These effects are exerted by multiple complementary mechanisms involving the modulation of p21 protein levels, inactivation of the PI3K/AKT pathway, and activation of the p53/p21 pathway 36,37, leading to synergistic anti-leukemic activity against *FLT3-ITD* AML. Our study provides a treatment option with high efficacy, low cost, and minimal side effects for patients with *FLT3-ITD* AML and maybe shows a survival advantage for FLT3-combination therapy in patients with *FLT3-ITD* R/R AML. Nevertheless, in future investigations, we intend to perform clinical studies to further corroborate our findings.

## Supplementary Material

Supplementary materials and methods, figures and tables.

## Abbreviations

AML: Acute myeloid leukemia
*FLT3-ITD*: FMS-like tyrosine kinase-internal tandem duplication
R/R AML: Relapsed/refractory Acute myeloid leukemia
TKI: Tyrosine kinase inhibitor
PI3K: Phosphatidylinositol-3-kinase
STAT5: Signal transducer and activator of transcription
AKT: Protein kinase B
MAPK: Mitogen-activated protein kinase
MEK: MAPK/ERK Kinase
hCD45: Human leukocyte common antigen 45
CDK2: Cyclin-dependent kinases 2
DMSO: Dimethyl sulfoxide
