# Supplementary material for: Mitoxantrone-liposome Sensitizes FLT3-ITD Acute Myeloid Leukemia to Gilteritinib Treatment
Source: J Cancer. 2025 Feb 28;16(6):1905–17. doi: 10.7150/jca.105557 (PMC11905413; doi:10.7150/jca.105557)
Supplement: Supplementary file 1 — Supplementary materials and methods, figures and tables. [file jcav16p1905s1.pdf]

Supplementary Information

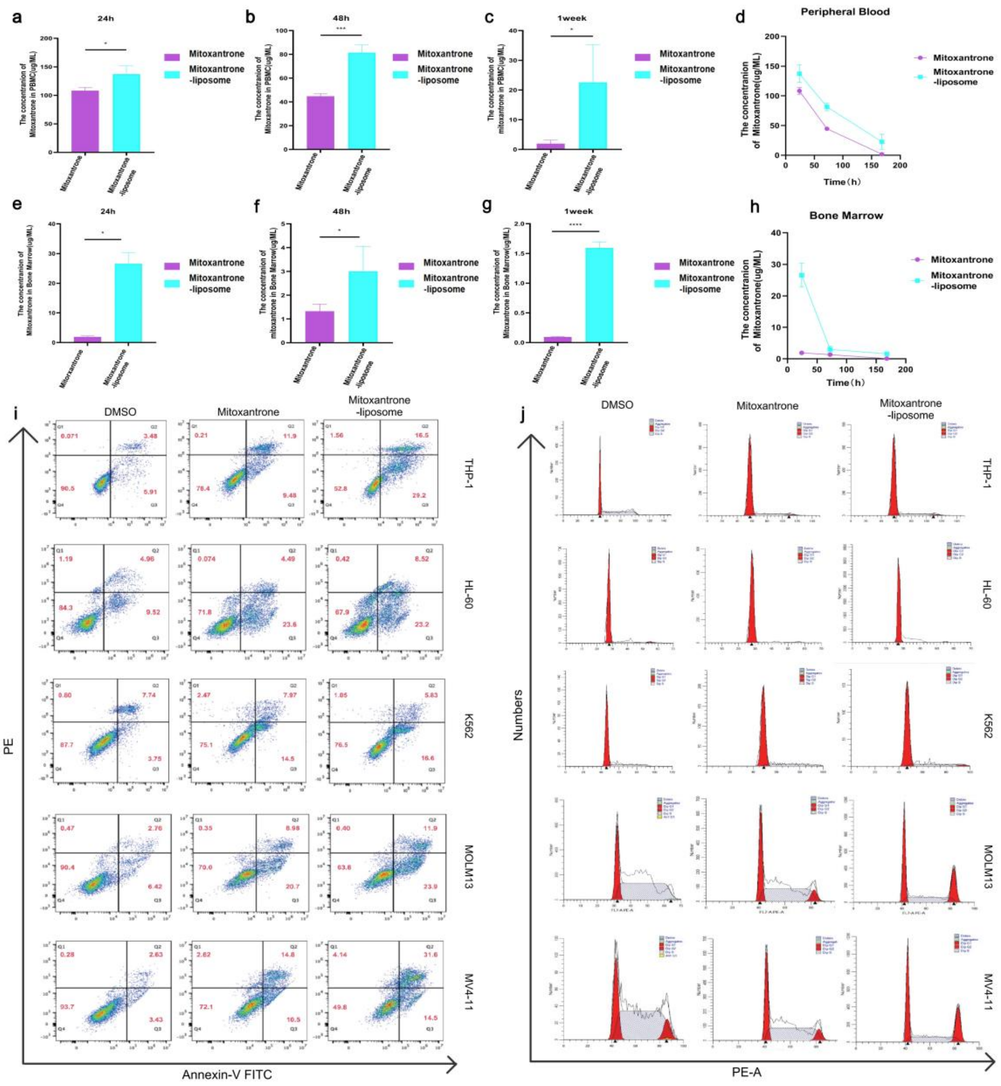

Figure. S1 Comparison of Mitoxantrone-liposome and Mitoxantrone. a-d. The concentrations of Mitoxantrone in peripheral blood in leukemia mice were measured in 24h, 72h and 1 week were by liquid chromatography. **e-h.** The concentrations of Mitoxantrone in bone marrow inleukemia mice were measured 24h, 72h and 1week. **i.** Apoptosis by flow cytometry in THP-1, HL-60, K562, MOLM-13, MV4-11 after 24h treatment (mitoxantrone-liposome (20 nM) and mitoxantrone treatment (20 nM)**)**. **j.** Cell cycle by flowcytometry in THP-1, HL-60, K562, MOLM-13, MV4-11 after 24h treatment (mitoxantrone-liposome (20 nM) and mitoxantrone treatment (20 nM)**)**.

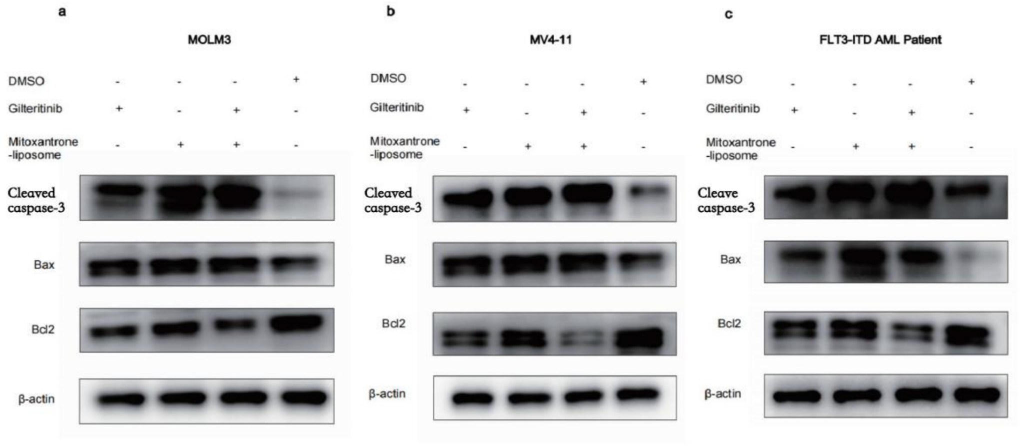

Figure. S2 Determination of apoptotic proteins. a-b. The protein expression level of Bax, Bcl2, Cleave-Caspase-3 were measured by western blotting after drug treatment (Gilteritinib: 25 nM, mitoxantrone-liposome: 20 nM, concentrations of combination durg: gilteritinib 20 nM, mitoxantrone-liposome 20 nM) in MOLM-13, MV4-11 and *FLT3-ITD* AML patient cells.

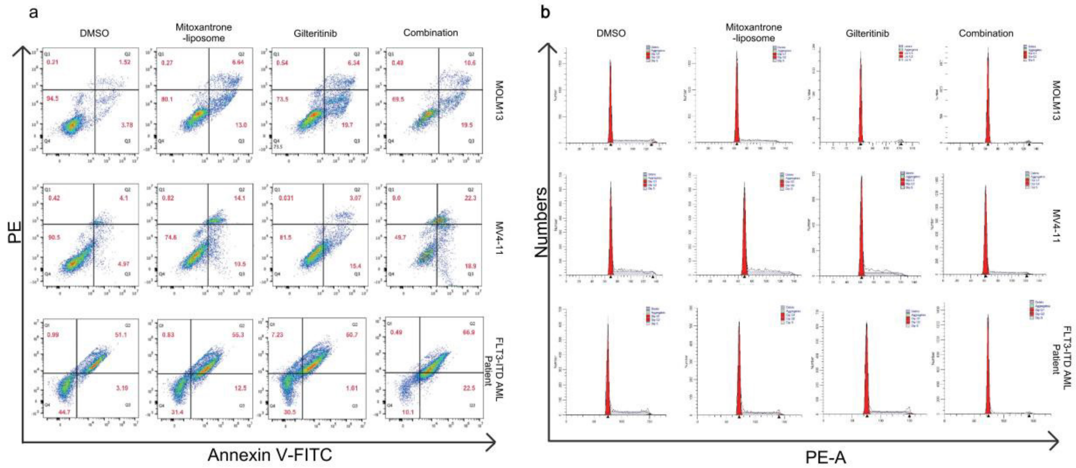

Figure. S3 Apoptosis and Cell cycle picture. a. Apoptosis by flow cytometry in MOLM-13 and MV4-11 cells and *FLT3-ITD* AML patient cells after 24h treatment. **b.** Cell cycle by treatment in MOLM-13 and MV4-11 cells and *FLT3-ITD* AML patient cells after 24h treatment. (Gilteritinib: 25 nM, mitoxantrone-liposome: 20 nM, concentrations of combination durg: gilteritinib 20 nM, mitoxantrone-liposome 20 nM)

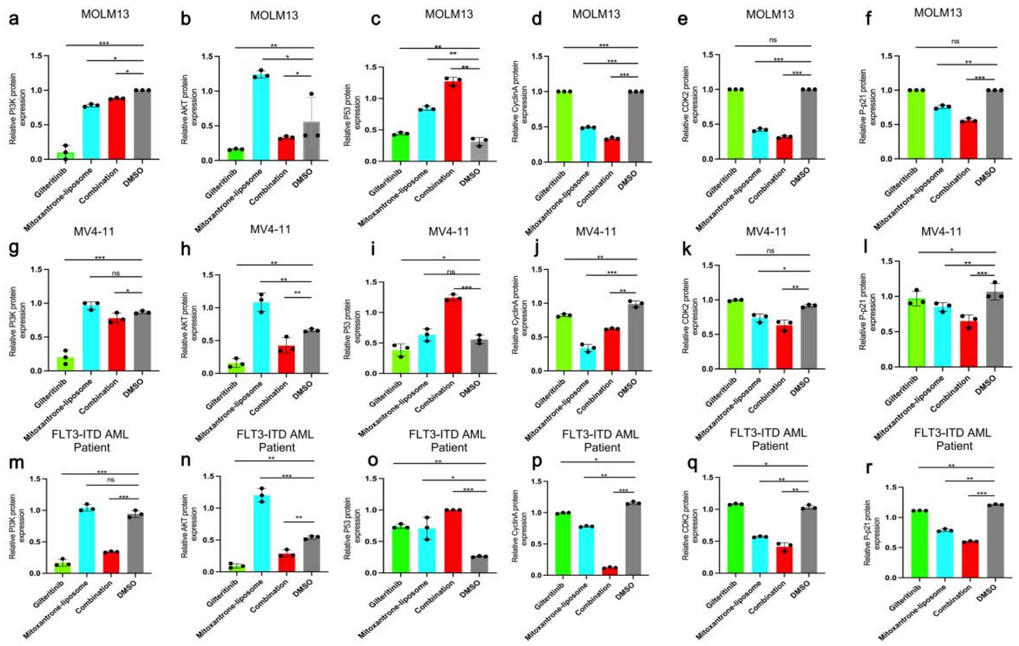

Figure. S4 Densitometry measurements aregraphed. The protein expression level of PI3K, AKT, P21, Phosphorylation P21, CyclinA/CDK2 were measured by western blotting after 12 h treatment in MOLM-13, MV4-11 and *FLT3-ITD* AML patient cells. (Gilteritinib: 25 nM, mitoxantrone-liposome: 20 nM, concentrations of combination durg: gilteritinib 20 nM, mitoxantrone-liposome 20 nM)

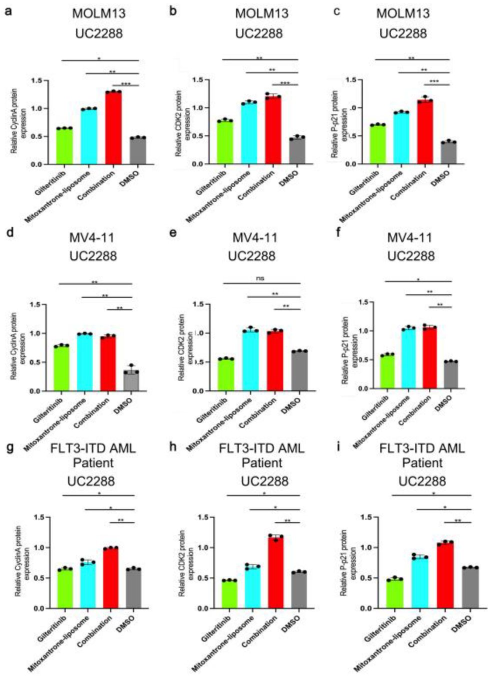

Figure. S5 Densitometry measurements aregraphed. The protein expression level of CDK2 were measured by western blotting after using drug and P21 inhibitor (UC2288) after 12 h in MOLM-13,

MV4-11 and *FLT3-ITD* AML patient cells. (Gilteritinib: 25 nM, mitoxantrone-liposome: 20 nM, concentrations of combination durg: gilteritinib 20 nM, mitoxantrone-liposome 20 nM, P21 inhibitor (UC2288): 10 nM).

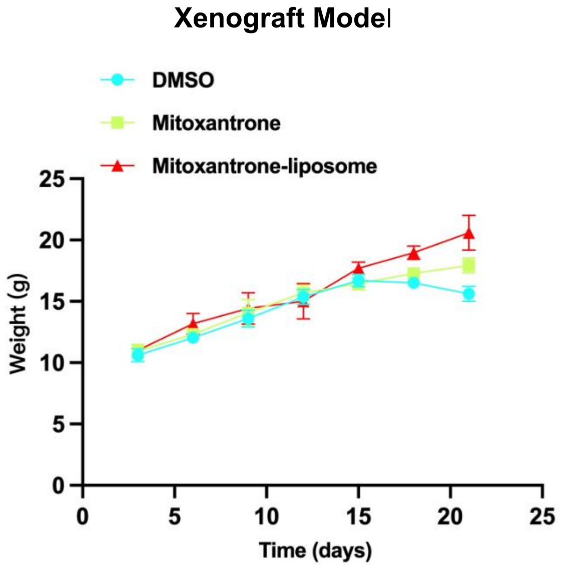

Figure. S6 Body weight changes after DMSO, Mitoxantrone , Mitoxantrone-liposome injections in a xenograft mouse model (vehicle (DMSO), mitoxantrone (5 mg/kg, d 7 injection), and mitoxantrone-liposome (5 mg/kg, d 7 injection)).

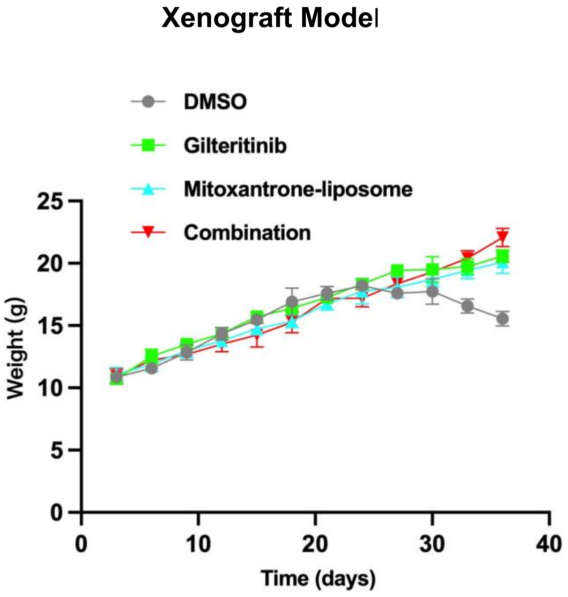

Figure. S7 Body weight changes after DMSO, Mitoxantrone, Mitoxantrone-liposome injections in a xenograft mouse model (vehicle (DMSO), mitoxantrone-liposome (5

mg/kg, d 1 injection), gilteritinib (30 mg/kg, 28 consecutive days, orally), and a combination of both drugs).

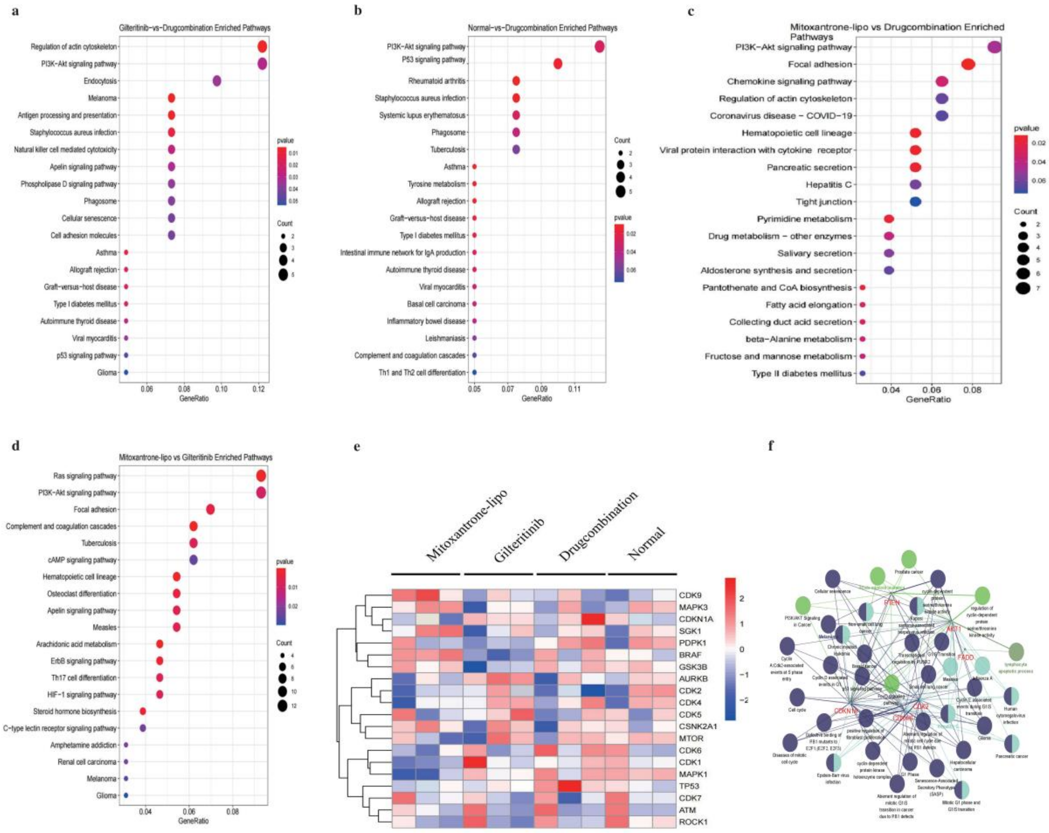

Figure. S8 a-d. Comparison of signaling pathways between single (DMSO, Gilteritinib, Mitoxantrone-liposome) and combination drugs in RNA-sequencing based on KEGG analysis. **e.** Heatmap representation of Average expression. **f.** Network map of gene and disease based on GO enrichment.

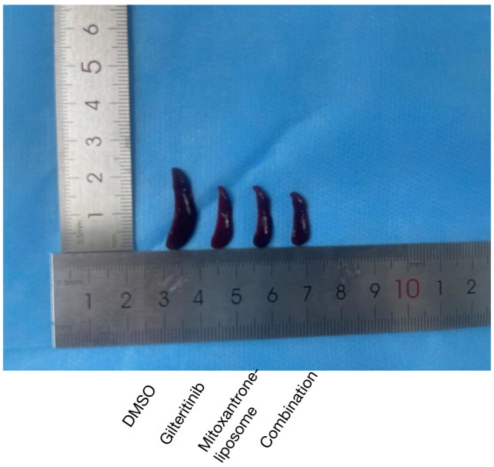

Figure. S9 The spleen size of DMSO, Gilteritinib, Mitoxantrone-liposome and Combination.

Table. S1 Combination Index value for Fig. 2

Combination Index (CI)		
MOLM13	Gilteritinib 34.76nM+Mitoxantrone-liposome 21.7nM	0.513
MV4-11	Gilteritinib 25.6nM+Mitoxantrone-liposome 23.49nM	0..46
FLT3-ITD AML Patient	Gilteritinib 21.56nM+Mitoxantrone-liposome 30.75nM	0.35
THP-1	Gilteritinib 600nM+Mitoxantrone-liposome 30.0nM	35.37 (>>1)
K562	Gilteritinib 1100nM+Mitoxantrone-liposome 30.1nM	46.72 (>>1)
HL60	Gilteritinib 700nM+Mitoxantrone-liposome 22.34nM	56.89 (>>1)

Table. S2 Patient characteristics of parimary AML patient samples.

Patients	Gender	Age(years)	Diease status	FAB subtype	Mutation Gene	FLT3 Mutation ratio
AML-1	Female	51	Newly diagnosed	M1	FLT3-ITD，NARS，PTPN11	56.25%
AML-2	Female	51	Newly diagnosed	M4	FLT3-ITD	73.83%
AML-3	Male	72	Newly diagnosed	M1	FLT3-ITD(high),DNMT3A,NPM1SF3B1	70.83%
AML-4	Female	51	Newly diagnosed	M2	FLT3-ITD,NPM1,DNMT3A	55.77%
AML-5	Male	72	Newly diagnosed	M3	FLT3-ITD(high)；AGTTTCCA；CEBPA	62.84%
AML-6	Male	24	Relapsed	M5	FLT3-ITD	44.35%
AML-7	Female	67	Newly diagnosed	MDS	FLT3(ITD)	46.39%
AML-8	male	24	Newly diagnosed	AML	FLT3-ITD	44.35%

Table. S3 The Flow sheet for specimen collection from patients with AML

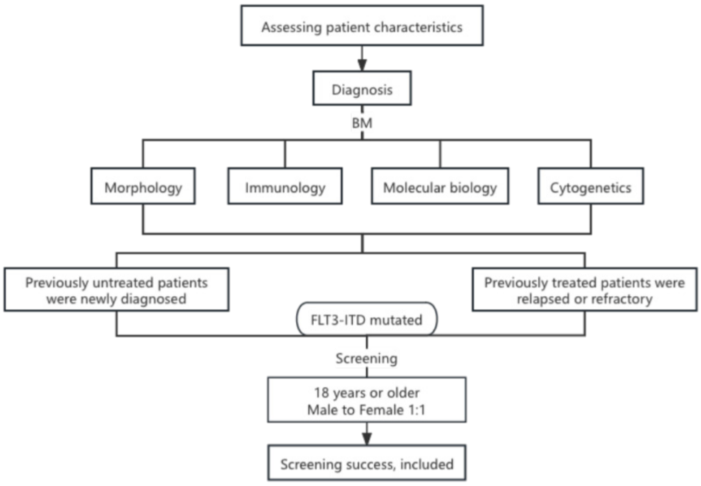

Table. S4 Primer list

Primers used for Real-Time qcr	
FLT3-Forward Primer	GAAACGGCCATCCCTAATT
FLT3-Reversr Primer	TTAGGGATAGGTGGAGGGATGAA
AKT-Forward Primer	GGACAAGGACGGGCACATTA
AKT-Reversr Primer	CGACCGCACATCATCTCGTA
CDK2-Forward Primer	GTACCTCCCCTGGATCGAAGAT
CDK2-Reversr Primer	CGAAATCCGCTTGTTAGGGTC
Caspase-3-Forward Primer	TGCTATTGTGAGGCGGTTGT
Caspase-3-Reversr Primer	TCACGGCCTGGGATTTCAAG
P53-Forward Primer	TGACACGCTTCCCTGGATTG
P53-Reversr Primer	GCTCGACGCTAGGATCTGAC
CyclinA-Forward Primer	CCTCGGTGTCCTACTTCAAATGT
CyclinA-Reversr Primer	TTCATCTTAGAGGCCACGAACAT

Materials and methods

RNA-sequencing (RNA-seq) analysis

Total RNA was isolated from single/combination drug and control MV4-11 cells using TRIzol® reagent.The ligated products were amplified by PCR, and the average insert length of the final cDNA library was 300±50 bp.Finally, 2×150 bp paired-end sequencing (PE150) was performed on illumina Novaseq™ 6000 (LC-Bio Technology CO., Ltd., Hangzhou, China) following the supplier's recommended protocol. Finally, 2×150 bp paired-end sequencing (PE150) was performed on illumina Novaseq™ 6000 (LC-Bio Technology CO., Ltd., Hangzhou, China) according to the protocol recommended by the supplier. Reads containing aptamer contamination, low-quality bases, and unidentified

bases were removed using FASTP. 10 sequences were also verified for quality using FASTP. Gene differential expression analysis was performed between two different groups (and between two samples) using DESeq2 software. Genes with a false discovery rate (FDR) of less than 0.05 and an absolute fold change of ≥2 were considered differentially expressed genes. Differentially expressed genes were then analyzed for GO function and KEGG pathway enrichment. The whole transcriptome sequencing of peripheral blood leukocytes was performed by Shanghai Tissuebank Diagnostics Co.Ltd.(China)

FLT3-ITD AML patients Inclusion criterial

1. Diagnosis of acute myeloid leukemia according to the WHO (2022) version.
2. Patients with FLT3-ITD mutations on genetic screening.
3. Informed consent explained to, understood by and signed by the patient or patient's guardian.

The inclusion process was as follows: A total of eight adult patients (18 years or older) diagnosed with AML between March 2022 and October 2022 were included in the study, with a male to female ratio of 1:1. They were divided into newly diagnosed AML (n = 7) and relapsed/refractory AML (n = 1). Eight bone marrow aspirates from age-matched healthy controls were also evaluated. Chart review was performed to extract patient data using the hospital system (Epic Systems, Chongqing Medical University). The study was approved by the review board of the Second Affiliated Hospital of Chongqing Medical University (**Table S2, Table S3**).
